# Homotypic Membrane Vesicle-Formulated VAN@^Δagr^MVs for Methicillin-Resistant *Staphylococcus aureus* Biofilm Clearance

**DOI:** 10.34133/bmr.0288

**Published:** 2025-12-09

**Authors:** Jianxiong Dou, Huagang Peng, Shu Li, Weilong Shang, Yi Yang, Xiaomei Hu, Li Tan, Zhen Hu, Yuting Wang, Feng Lin, Qiwen Hu, Chuan Xiao, Xiaoran Jiang, Ming Li, Xiancai Rao

**Affiliations:** Department of Microbiology, College of Basic Medical Sciences, Army Medical University, Key Laboratory of Microbial Engineering under the Educational Committee in Chongqing, Chongqing 400038, China.

## Abstract

Multidrug-resistant (MDR) pathogens such as methicillin-resistant *Staphylococcus aureus* (MRSA) pose a substantial challenge to global public health, particularly because of chronic and persistent infections associated with bacterial biofilms, which call for safe and innovative therapeutic strategies. Here, we present a novel antibiofilm system inspired by the preferential uptake properties of isogenous bacterial membrane vesicles (MVs). This system employs vancomycin (VAN) for bacterial killing, while MVs act as delivery vehicles to increase VAN penetration into biofilms. VAN@^Δagr^MVs demonstrated sustained drug release and improved VAN accessibility within biofilms. Treatment with VAN@^Δagr^MVs considerably reduced the number of planktonic MRSA strain USA300 cells and effectively eradicated MRSA biofilms in vitro. RNA sequencing revealed substantial alterations in genes associated with bacterial cell wall biosynthesis, global regulators, virulence factors, and biofilm formation. Treatment with VAN@^Δagr^MVs substantially reduced the MRSA burden within biofilms in vivo. Safety evaluation demonstrated the avirulent properties of the VAN@^Δagr^MVs, highlighting its potential for clinical application. Overall, this study offers a promising alternative for MRSA biofilm eradication, providing a viable strategy to combat chronic infections caused by MDR biofilm-forming pathogens.

## Introduction

The globalization of multidrug-resistant pathogens, including methicillin-resistant *Staphylococcus aureus* (MRSA) and carbapenem-resistant *Pseudomonas aeruginosa* [1], has been well documented since the late 20th century, posing increasing challenges for infectious disease control [2]. Notably, MRSA-associated deaths have increased globally (from 261,000 related deaths in 1990 to 550,000 in 2021), underscoring the urgent need for novel therapeutics to control MRSA fatalities [3].

As a gram-positive pathogen, MRSA colonizes diverse human anatomical sites, causing a broad spectrum of infections [4]. MRSA’s capacity to form highly structured, syntrophic mono- and mixed-species biofilms further amplifies drug resistance, driving elevated morbidity and mortality [5]. Bacterial biofilm formation is a complex, multistage process. Moormeier and Bayles [6] delineated 5 key stages: attachment, multiplication, exodus, maturation, and dispersal, each governed by intricate regulatory networks [7], which are orchestrated by quorum sensing (QS) systems (Agr and LuxS/AI-2), 2-component signal transduction systems (SaeRS and WalKR), and staphylococcal accessory regulator (Sar) family regulators (SarA and MgrA) [8]. Undoubtedly, deciphering staphylococcal biofilm biology and regulation is pivotal for developing therapies to disrupt established biofilms in clinical settings [7]. However, highly effective treatments for MRSA biofilms remain an unmet medical need.

Extensive efforts have been devoted to developing effective strategies against bacterial biofilm infections. Current antibiotic alternatives include QS inhibitors, bacteriophages, endolysins, lysozymes, antimicrobial peptides, and surfactants [9–11]. However, these bioactive agents are easily inactivated by microbial extracellular matrix (ECM) and penetrate poorly into the established biofilms, limiting their therapeutic efficacy [12]. Therefore, improved delivery systems for antibiofilm agents are urgently needed.

Vancomycin (VAN) has been considered as a last-resort drug to treat severe MRSA infections, whereas bacterial biofilms are commonly inaccessible for free VAN [13]. Several VAN-loaded nanoparticles have been developed for biofilm eradication [14,15]. Kang et al. [14] fabricated a magnetic nanoparticle loaded with rhamnolipid (RL) and VAN (Vanc/RL-Ag@Fe_3_O_4_) and found a magnetic field promoted the anti-biofilm effect of VAN-loaded particles by facilitating its penetration into the bottom layers of subgingival biofilms formed with *Streptococcus oralis*, *Streptococcus sanguinis*, *Porphyromonas gingivalis*, *Actinomyces naeslundii*, and *Fusobacterium nucleatum*. Mu et al. [15] used the thin-film hydration method to prepare VAN-loaded microbubbles (VAN-MBs), and they revealed that VAN-MBs penetrated deeper into MRSA biofilms compared with free VAN. However, the application of these VAN-loaded materials needs specific equipment assistance, such as magnetic field for particle penetration and ultrasound-targeted microbubble destruction.

Membrane vesicles (MVs) are spherical nanoparticles that are naturally secreted by bacteria during growth [16]. MVs-based vehicles have emerged as promising drug delivery systems due to their excellent biocompatibility and high surface-to-volume ratio [17] and have been applied in various fields, such as cancer diagnosis and immunotherapy [18,19]. Notably, MVs demonstrate homotypic targeting capabilities. Peng et al. [20] reported that *Escherichia coli*-derived MVs are preferentially internalized by *E. coli* but not gram-positive bacteria such as *S. aureus*. Moreover, coating nanoparticles with a hybrid membrane of red blood cells and *E. coli* MVs enhanced *E. coli*-targeting efficiency by 2.8-fold compared with uncoated controls [21]. MVs derived from gram-negative bacteria such as *E. coli* have been extensively studied to carry antibiotics [22]. However, gram-negative bacterial MVs often encapsulate lipopolysaccharide (LPS), an outer membrane-bounded endotoxin contributing to bacterial pathogenicity [16]. By contrast, gram-positive bacteria such as *S. aureus* lack LPS, and their MVs have good biocompatibility [16]. Therefore, we speculated that isogenic MVs derived from *S. aureus* would exhibit preferential targeting and enhanced penetration into MRSA biofilms, thereby improving the biofilm eradication efficiency of MV-encapsulated antimicrobial agents. To test this hypothesis, we selected VAN as a therapeutic payload and used *S. aureus* strain RN4220-Δ*agr* as a host for the production of attenuated ^Δagr^MVs [16]. The accessory gene regulator (Agr) is a major QS system that regulates gene expression of virulence factors in *S. aureus* [4,8]*.* Compared with MVs derived from the wild-type *S. aureus* strain, ^Δagr^MVs present low toxicity and are safe for delivery vehicles [16]. VAN-loaded ^Δagr^MVs (VAN@^Δagr^MVs) were prepared and utilized to treat MRSA in a planktonic state and in structured biofilms. The results demonstrated that VAN@^Δagr^MVs effectively eliminate MRSA biofilms both in vitro and in vivo, highlighting their potential as a novel therapeutic approach against biofilm infections.

## Materials and Methods

### Bacterial strain and culture

The *S. aureus* strain RN4220-Δ*agr* was generated previously [16], and the MRSA strain USA300 (FPR3757) was kindly provided by Dr. Min Li (Shanghai Jiao Tong University, China). *S. aureus* strains were cultured in brain heart infusion (BHI) medium (Oxoid, UK) or BHI agar (BHIA) at 37 °C.

### Preparation of ^Δagr^MVs and VAN@^Δagr^MVs

^Δagr^MVs were prepared from the culture supernatant of *S. aureus* strain RN4220-Δ*agr* as previously described [23]. Briefly, a single colony grown on BHIA was picked and inoculated into fresh BHI broth and cultivated overnight at 37 °C. Next, the culture mixture was diluted 1:100 in 300 ml of fresh BHI broth and cultured at 37 °C for 12 h. The supernatant containing ^Δagr^MVs was collected by centrifugation at 6,000 ×*g* for 10 min, followed by centrifugation at 10,000 ×*g* for 10 min and filtration through a 0.22-μm filter (Merck Millipore, USA) to remove dead cells and cellular debris. The filtered solution was concentrated by ultrafiltration through a 100-kDa fiber membrane column (GE Healthcare, USA) to enrich the ^Δagr^MVs. After ultracentrifugation at 200,000 ×*g* for 3 h with a rotor (HITACHI, Japan), the ^Δagr^MVs pellets were resuspended in 4 ml of 50% (v/v) Optiprep density gradient solution (Alere Technologies AS, Norway), followed by the addition of 2 ml of 40% (v/v), 2 ml of 20% (v/v), and 1.5 ml of 10% (v/v) Optiprep solution, in that order. After centrifugation at 200,000 ×*g* for 3 h at 4 °C, 5 fractions from top to bottom were transferred into sterile tubes. SDS-PAGE (Servicebio, China) was performed to characterize the fractions. The ^Δagr^MVs located between the 20% and 40% Optiprep solutions were extracted and concentrated via an ultrafiltration tube (Millipore). The pellets were dissolved in phosphate-buffered saline (PBS, pH 7.2), followed by filtration through a 0.22-μm syringe filter (Millipore). The sterility of ^Δagr^MVs sample was confirmed by culturing in BHI medium, and the sterile samples were stored at −80 °C.

VAN@^Δagr^MVs were prepared as previously described [24,25]. In brief, VAN (Sigma-Aldrich, USA) and the prepared ^Δagr^MVs were mixed at a mass ratio of 2:1 as previously described [25], and the mixture was then subjected to sonication via an UP-50H ultrasonic cell disruptor (Hielscher, Germany) at 30% power for 6 cycles with a 4-s pulse and a 2-s pause. After that, the mixture was incubated at 37 °C for 1 h to allow ^Δagr^MVs to be restored. Ultrafiltration was performed via an Amicon ultracentrifugation filter (molecular weight cutoff = 100 kDa; GE Healthcare, UK) to remove free VAN. The purified VAN@^Δagr^MVs were aseptically filtered and stored at −80 °C.

### Characterization of the ^Δagr^MVs and VAN@^Δagr^MVs

The samples (^Δagr^MVs and VAN@^Δagr^MVs) were dropped onto copper grids, allowed to sediment naturally for 15 min, negatively stained with 2% (v/v) phosphotungstic acid for 15 s, and dried for 1 h. Transmission electron microscopy (TEM; HT7700, Hitachi, Japan) was used to observe the morphology of the ^Δagr^MVs and VAN@^Δagr^MVs. The particle size distributions and zeta potentials of the ^Δagr^MVs and VAN@^Δagr^MVs were detected via a dynamic light scattering (DLS) Nanoparticle Size Analyzer (Zatasize Nano ZSP, Malvern, USA).

### VAN encapsulation and release

The high-performance liquid chromatography (HPLC) was carried out to quantify VAN@^Δagr^MVs as previously reported [25]. Briefly, freshly prepared VAN@^Δagr^MVs were added to a dialysis bag (100 kDa, Ruiswbio, USA), immersed in 100 ml of PBS, and incubated at 37 °C with magnetic stirring. Aliquots (1 ml of PBS per time point) were collected and analyzed via HPLC. The aliquot was mixed with an appropriate volume of acetonitrile in a sterile tube. After sonication, the supernatant was obtained by centrifugation at 18,000 ×*g* for 10 min, filtered through a 0.22-μm filter (Millipore), and transferred to an HPLC autosampler. Approximately 1 ml of each sample was loaded into an HPLC system (Agilent 1260, Agilent Technologies, CA) equipped with a C18 column (extended-C18, 250 mm × 4.6 mm, 5 μm, 100 Å, Agilent). A mobile phase (KH_2_PO_4_:acetonitrile, 90.5:9.5, v/v, pH 3.2) was used at a flow rate of 1 ml/min. VAN elution was monitored at 236 nm, and a standard curve with concentrations of VAN ranging from 3.125 to 50 μg/ml was established via OpenLAB CDS ChemStation Edition software (Fig. S1). The release profile of VAN from VAN@^Δagr^MV particles was determined in PBS at pH 7.4. VAN loading and encapsulation efficiencies were calculated using the following formulas:

VAN loading efficiency (%) = weight of VAN in VAN@^Δagr^MVs/weight of the total VAN@^Δagr^MVs × 100%

VAN encapsulation efficiency (%) = weight of VAN in VAN@^Δagr^MVs/weight of the total amount of VAN in preparation of VAN@^Δagr^MVs× 100%.

### Bactericidal effect of VAN@^Δagr^MVs on MRSA

The antibacterial effect of VAN@^Δagr^MVs on planktonic MRSA was determined as described previously [26]. Briefly, MRSA USA300 was cultured overnight. The next day, the culture was diluted to 1 × 10^6^ colony-forming units (CFU)/ml with PBS, and 100 μl of the MRSA suspension was transferred to each well of a 96-well plate. Then, 100 μl of PBS, VAN (20 μg/ml), ^Δagr^MVs (100 μg/ml), or VAN@^Δagr^MVs (100 μg/ml) was added to each well. After 0, 6, and 24 h of incubation, 10 μl of each sample was collected for bacterial counting. Bacterial cells treated with diverse concentrations of VAN@^Δagr^MVs (0, 6.25, 12.5, 25, and 50 μg/ml) were also analyzed, and CFU counts were determined at 12 and 24 h. For confocal microscopy, MRSA USA300 cells treated for 6 h were collected and stained with a LIVE/DEAD Cell Staining Kit (Beyotime, China), followed by observation under a super-resolution laser scanning confocal microscope (LSM880, Zeiss, Germany).

### MVs-mediated penetration of VAN into MRSA biofilms

Overnight-cultured MRSA USA300 was diluted to 1 × 10^6^ CFU/ml with PBS, and 1 ml of bacterial suspension was cultivated in a confocal dish (In Vitro Scientific, China) for 24 h to form biofilms. After removal of the culture supernatant, the biofilm was washed twice with PBS. Then, ^Δagr^MVs (10 μg/ml) and VAN@^Δagr^MVs (10 μg/ml) prestained with PKH26 (Umibio, China) were added separately, and the confocal dish was incubated at 37 °C for 90 min. The supernatant was removed, and the biofilms were fixed with 4% (v/v) paraformaldehyde (Biosharp, China) for 15 min, followed by staining with 20 μM SYTO9 (APExBIO, USA) and Calcofluor (5 μl/ml, Sigma-Aldrich, USA) for 20 min before visualization under an LSM880 confocal microscope.

VAN penetration was also determined with fluorescein isothiocyanate (FITC)-labeled VAN (FITC-VAN). FITC-VAN (Ruixibio, Xi’an, China) was used to prepare FITC-VAN@^Δagr^MVs. MRSA USA300 biofilms were established and treated with FITC-VAN (10 μg/ml) or FITC-VAN@^Δagr^MVs (10 μg/ml). After fixation with 4% (v/v) paraformaldehyde, the biofilm matrix was stained with Calcofluor (5 μl/ml) for 20 min, and the fluorescence in each sample was observed via a confocal microscope (LSM880).

### Elimination of MRSA biofilms with VAN@^Δagr^MVs in vitro

The overnight-cultured *S. aureus* USA300 was prepared as a 1 × 10^6^ CFU/ml suspension with BHI. Next, 200 μl of bacterial suspension was added to the wells of a 96-well plate and cultured at 37 °C for 24 h to form biofilms. The culture supernatant was discarded, and the biofilm was washed twice with PBS. The biofilms were subsequently treated with PBS, VAN (10 μg/ml), ^Δagr^MVs (50 μg/ml), or VAN@^Δagr^MVs (50 μg/ml) for 24 h at 37 °C. Different concentrations of VAN@^Δagr^MVs (0, 10, 20, and 40 μg/ml) were also used to treat the established MRSA biofilms. After treatment, the bacterial cells within the biofilms were counted via a plate dilution assay as previously described [25]. Additionally, crystal violet staining (Solarbio, China) was performed to detect the treated biofilms by measuring the OD values at 595 nm via a microplate reader (Thermo Fisher Scientific, USA).

### Scanning electron microscopy

The morphological features of MRSA biofilms treated with PBS, VAN (10 μg/ml), ^Δagr^MVs (50 μg/ml), or VAN@^Δagr^MVs (50 μg/ml) were observed via scanning electron microscopy (SEM). MRSA biofilms formed on coverslips (CITOTEST, China) were fixed with glutaraldehyde (Boer, China) overnight at 4 °C and then sequentially dehydrated in 50% (v/v), 70%, 80%, 90%, and 100% ethanol, followed by 100% tert-butyl alcohol. Finally, the biofilm samples were observed under a scanning electron microscope (ZEISS-Crossbeam 340, Germany).

### RNA-seq analysis

MRSA USA300 was cultured in BHI medium to the mid-log phase, and the cells were pelleted, washed, and diluted to 1 × 10^6^ CFU/ml with BHI. Next, 500 μl of bacterial suspension was added to the wells of a 24-well plate and cultured at 37 °C for 24 h to form biofilms. After supernatant removal, 100 μl of PBS, VAN (10 μg/ml), or VAN@^Δagr^MVs (50 μg/ml; equivalent to individual VAN content) was added and incubated with the biofilm for 3 h at 37 °C. After treatment, total bacterial RNA was extracted via an RNAprep Pure Cell/Bacteria Kit (TianGen, China). After quality checking, the total RNA was subjected to RNA sequencing (RNA-seq) at Novogene Co., Ltd. (Beijing). Bacterial rRNA was removed via a Ribo-Zero Gold Kit (Illumina), and RNA-seq libraries were generated via an Illumina TruSeq RNA Library Prep Kit. Sequencing was performed on an Illumina HiSeq NovaSeq-PE150 system. Each experimental group included 3 biological replicates. Differentially expressed genes (DEGs) were analyzed via the DESeq2 package with thresholds of |fold change| > 1.5 and adjusted *P* value < 0.05. RNA-seq data were submitted and deposited in the Gene Expression Omnibus (GEO) datasets for reference.

### Elimination of MRSA biofilms in vivo

Female BALB/c mice (aged 6 to 8 weeks) were purchased from Chongqing Byrness Weil Biotechnology Co., Ltd. (Chongqing, China). The mice were housed at room temperature with a 12-h light/12-h dark cycle. All animals had free access to food and water throughout the experiment. The animal studies were approved by the Laboratory Animal Welfare and Ethics Committee of Army Medical University, and the animal handling procedures followed the guidelines set by the Animal Care Committee, Army Medical University (Protocol no. AMUWEC2020735).

The 6-mm silicone sheets (Biosharp, Canada) were sterilized with 75% (v/v) alcohol for 1 h, followed by ultraviolet light irradiation on both sides (0.5 h each), and then placed into the wells of a 24-well plate. The overnight-cultured MRSA USA300 was diluted to 1 × 10^6^ CFU/ml with BHI and added to the plate wells (500 μl per well). The bacteria were cultured at 37 °C for 24 h. BALB/c mice were anesthetized, and the hair from the back and flank was removed. The skin on mouse back was cut and the silicone sheet with established MRSA biofilms was implanted subcutaneously. The silicone piece was fixed with medical glue (3 M Vetbond Tissue Adhesive, 3 M, USA) and the wound was covered with a 3 M Tegaderm film (3 M, USA). The model mice were randomly divided into 5 groups (*n* = 15 for each). A total of 50 μl of PBS, VAN (40 μg/ml), ^Δagr^MVs (200 μg/ml), or VAN@^Δagr^MVs (200 μg/ml) was given 4 times on day 1 (12 h), day 2 (36 h), day 3 (60 h), and day 5 (108 h) after biofilm implantation. The uninfected mice served as a normal control.

On days 2, 4, and 7, 5 mice of each group were sacrificed. The murine blood samples (*n* = 3) were collected for determination of inflammatory factors via an enzyme-linked immunosorbent assay (ELISA) kit (UPingBio, China). The infected tissues around the biofilm were taken and fixed with 4% (v/v) paraformaldehyde. Histopathological examination was performed via hematoxylin and eosin (H&E) staining. Moreover, the silicone sheets were obtained from murine wounds and subjected to bacterial counting via the broth dilution method as previously described [5,25]. Briefly, the removed silicone film was immersed in 1 ml of sterile PBS, and the bacteria on the film were dispersed by sonication for 1.5 min with repeated cycles of 2-s pulses and 2-s pauses. The sample was then serially diluted 10-fold with sterile PBS, and 10 μl of the sample was spread on a BHI plate and cultured at 37 °C overnight. The number of colonies that grew on the plate was calculated.

### Biosafety assay for VAN@^Δagr^MVs in vitro and in vivo

RAW264.7 and A549 cells were used to assess the cytotoxicity of VAN@^Δagr^MVs via the 3-[4,5-dimethylthiazol-2-yl]-2,5-diphenyltetrazolium bromide assay as previously described [27]. In brief, RAW264.7 macrophages or A549 cells were seeded in 96-well plates (2 × 10^3^ cells/well) and cultured at 37 °C for 24 h with 5% (v/v) CO_2_. The culture medium was replaced with fresh medium supplemented with various concentrations of VAN@^Δagr^MVs (0, 10, 20, 30, 40, and 50 μg/ml). Cell viability was determined 24 h posttreatment via a Cell Counting Kit (Zeta Life, USA). In addition, RAW264.7 and A549 cells treated with 50 μg/ml VAN@^Δagr^MVs for 24 h were stained with a LIVE/DEAD Cell Staining Kit (Beyotime, China) and visualized under an LSM880 microscope.

The mouse abdominal infection model (*n* = 3 per group) was generated with the MRSA strain USA300 as previously described [27]. Mice were then treated with PBS, ^Δagr^MVs (6 mg/kg), or VAN@^Δagr^MVs (6 mg/kg). After 24 h of treatment, the MRSA-infected mice were sacrificed, and their organs (heart, liver, spleen, lungs, and kidneys) were harvested and fixed for histological analysis with H&E staining. Murine serum IL-6 and TNF-α levels at 6 h posttreatment were determined via an ELISA kit (MEIMIAN).

### Statistical analysis

All experiments were repeated at least 3 times, and the data are expressed as the mean ± standard deviation (SD) or standard error of the mean. Statistical comparisons were performed via the GraphPad Prism 9.5 program (GraphPad Software, USA). Student’s *t* test, 1-way, or 2-way analysis of variance (ANOVA) was used to analyze differences among groups. A *P* value less than 0.05 was considered statistically significant.

## Results and Discussion

### Preparation of *S. aureus* MVs-derived nanoparticles

**Fig. 1.**
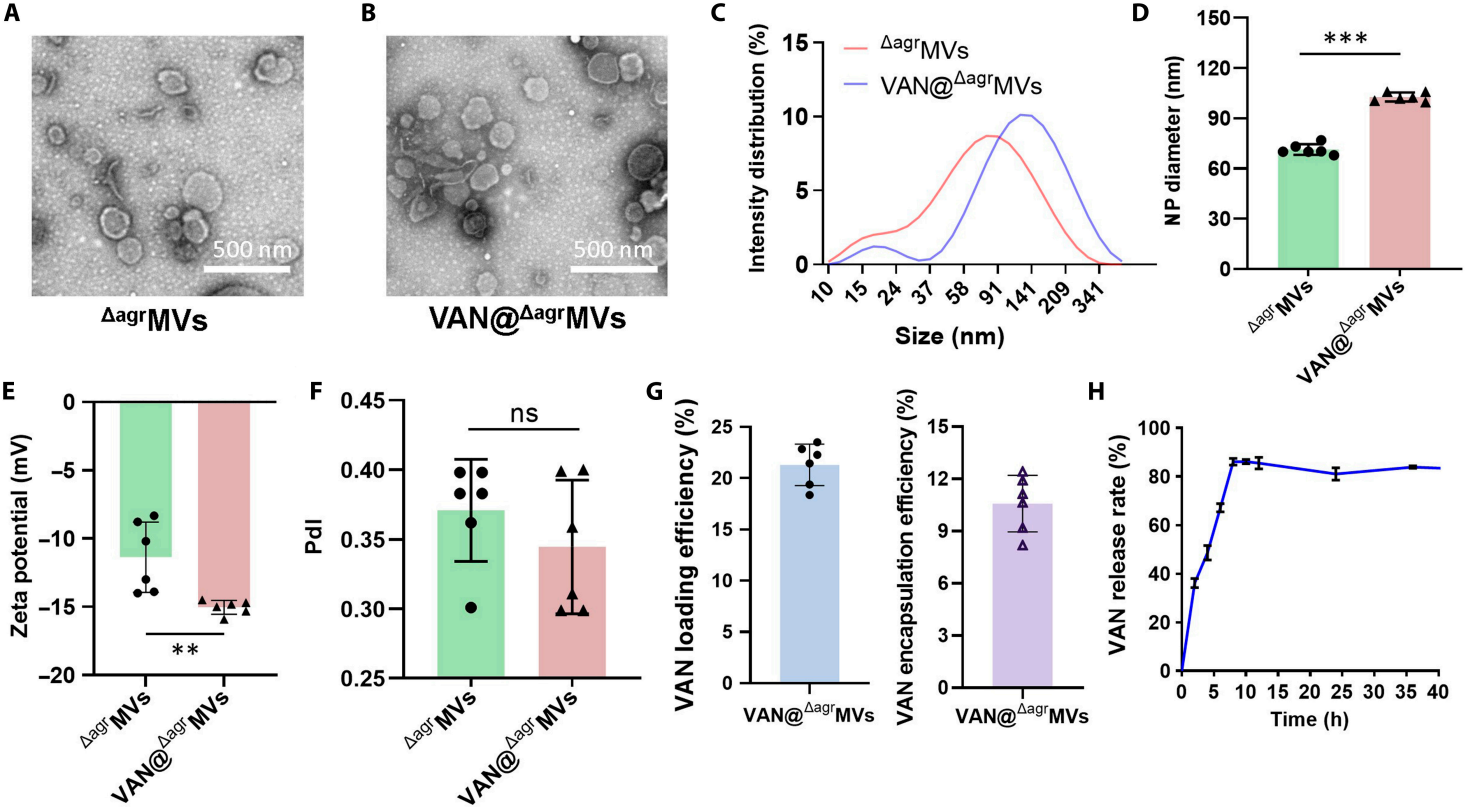
Characterization of ^Δagr^MVs and VAN@^Δagr^MVs. (A) Morphology of the ^Δagr^MVs observed via TEM (scale bar: 500 nm). (B) Morphology of VAN@^Δagr^MVs observed via TEM. (C) Particle size distributions of ^Δagr^MVs and VAN@^Δagr^MVs detected via DLS. (D) Nanoparticle (NP) diameter variation of ^Δagr^MVs and VAN@^Δagr^MVs measured via DLS. (E) Membrane surface potential of ^Δagr^MVs and VAN@^Δagr^MVs measured via DLS. (F) Polydispersity index (PDI) values of ^Δagr^MVs and VAN@^Δagr^MVs detected via DLS. (G) The drug loading and encapsulation efficiencies of VAN@^Δagr^MVs. (H) VAN release from VAN@^Δagr^MVs quantified by HPLC. The data are shown as the mean ± SD (*n* = 6). Statistical significance was calculated by Student’s *t* test, **P < 0.01, ***P < 0.001, and ns represents no significance.

Given that *S. aureus* MVs can encapsulate toxic cargo [28], we prepared MVs from an attenuated RN4220-Δ*agr* strain in which the entire virulence-regulatory *agr* locus was deleted [16]. TEM revealed that the ^Δagr^MVs exhibited a spherical morphology with characteristic bilayer membrane structures (Fig. 1A). VAN-loaded ^Δagr^MVs (VAN@^Δagr^MVs) were prepared via ultrasonication of a VAN/^Δagr^MVs mixture at a 2:1 mass ratio as described previously [25]. TEM imaging revealed that the morphology of VAN@^Δagr^MVs was similar to that of empty ^Δagr^MVs (Fig. 1B). To assess potential size changes following drug loading, we performed DLS analysis, a well-established method for determining hydrodynamic particle size [29]. The results revealed that the mean diameter of VAN@^Δagr^MVs (102.7 nm) was substantially greater than that of empty ^Δagr^MVs (71.4 nm) (Fig. 1C and D and Fig. S2). Such size variations are known to occur following the encapsulation of exogenous substances (e.g., calcium ions or SiO_2_ nanoparticles), genetic modifications, or antibiotic exposure [23,30]. The increase in size may facilitate the penetration of VAN@^Δagr^MVs into certain tissues or their internalization by immune cells; however, these potential effects warrant further investigation.

DLS determination also revealed that the zeta potential shifted from −11.37 mV (^Δagr^MVs) to −15.03 mV (VAN@^Δagr^MVs) (Fig. 1E), suggesting improved colloidal stability of *S. aureus* ^Δagr^MVs after VAN loading. Polydispersity index (PDI) analysis revealed that VAN@^Δagr^MVs exhibited similar size homogeneity compared to empty vesicles (Fig. 1F), further supporting their potential as drug carriers. The average drug loading efficiency (21.17%) and encapsulation rate (10.58%) of VAN@^Δagr^MVs were also determined (Fig. 1G). Moreover, HPLC analysis revealed sustained VAN release kinetics, with approximately 86.1% of the encapsulated drug being released within 8 h under neutral conditions (Fig. 1H). Collectively, these results demonstrate that VAN@^Δagr^MVs maintain the structural characteristics of native MVs while exhibiting favorable drug delivery properties, including a sustained release capability.

### Antibacterial activity of VAN@^Δagr^MVs against planktonic *S. aureus* in vitro

**Fig. 2.**
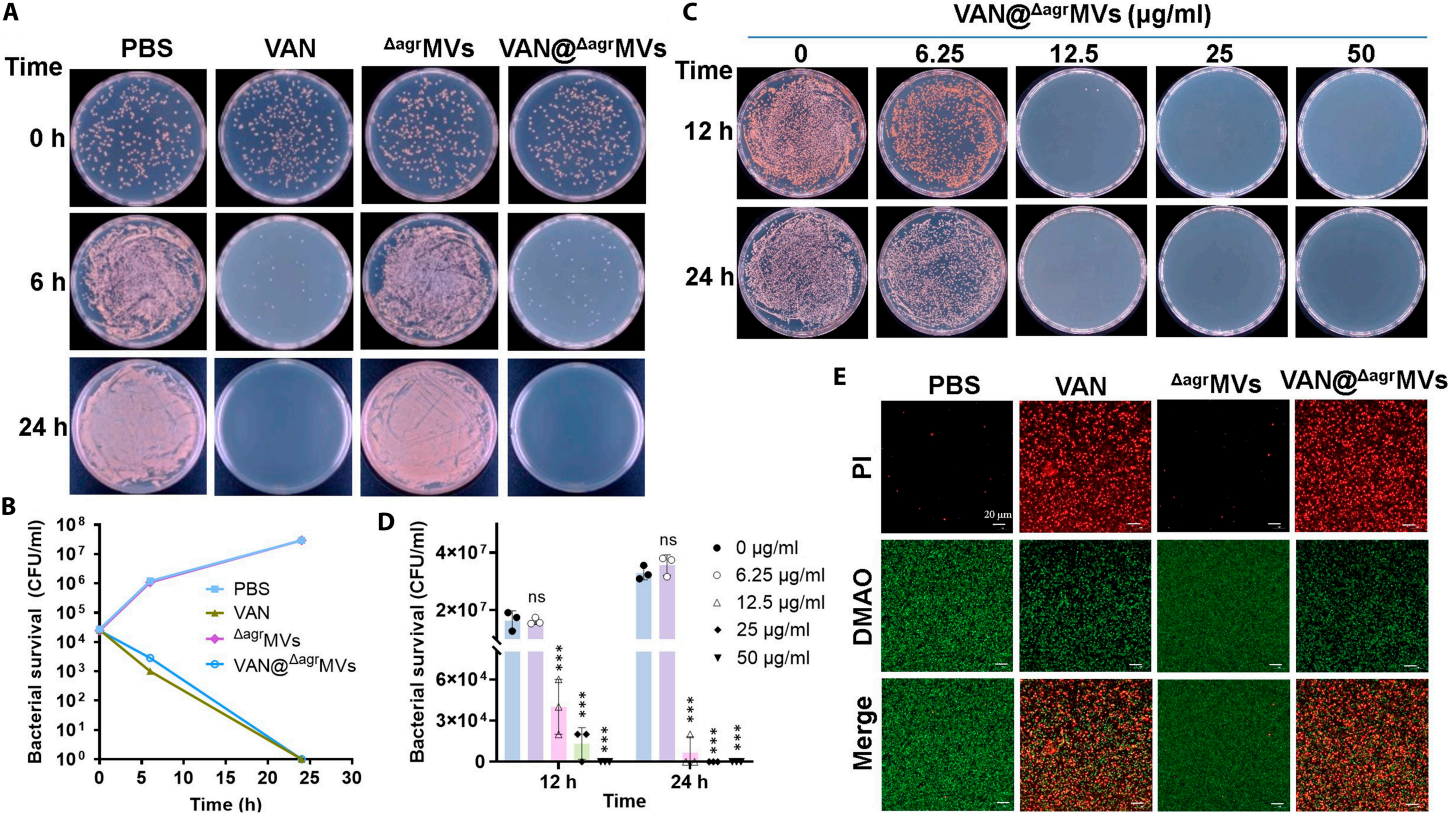
In vitro antibacterial activity of VAN@^Δagr^MVs. (A) Survivals of MRSA USA300 after treatment with PBS, free VAN (10 μg/ml), ^Δagr^MVs (50 μg/ml), or VAN@^Δagr^MVs (50 μg/ml) at 0, 6, and 24 h, respectively. (B) Bacterial counts from panel (A). (C) Survivals of MRSA USA300 treated with various concentrations of VAN@^Δagr^MVs for 12 and 24 h. (D) Bacterial survival counts from panel (C). (E) CLSM imaging of MRSA USA300 treated with PBS, free VAN (10 μg/ml), ^Δagr^MVs (50 μg/ml), or VAN@^Δagr^MVs (50 μg/ml) for 6 h. Bacterial cells were stained with a LIVE/DEAD cell staining kit and observed under a confocal microscope. Green fluorescence represents live bacteria, whereas the red fluorescence indicates dead cells (scale bar: 20 μm). The data are shown as the mean ± standard error of the mean. Statistical significance was measured by 1-way ANOVA, ***P < 0.001, and ns represents no significance.

Given the slow release profile of VAN from VAN@^Δagr^MVs, we hypothesized that VAN@^Δagr^MVs could exhibit antibacterial activity in vitro. To this end, we performed agar plate counting as previously described [31]. Mid-log phase *S. aureus* USA300 cultures (OD_600_ = 0.5) were washed and resuspended in PBS, after which approximately 1 × 10^6^ CFU of bacteria were treated with 10 μg/ml of VAN, 50 μg/ml of ^Δagr^MVs, or 50 μg/ml of VAN@^Δagr^MVs. Bacterial viability was assessed at 0, 6, and 24 h posttreatment. Compared with the PBS control, both free VAN and VAN@^Δagr^MVs exhibited bactericidal activity, whereas the ^Δagr^MVs alone had no intrinsic antibacterial effects (Fig. 2A). Time-dependent killing kinetic analysis further confirmed the comparable anti-staphylococcal activity of VAN@^Δagr^MVs and free VAN (Fig. 2B). These results are consistent with previous findings that MVs derived from the *Lacticaseibacillus casei* strain BL23 lack direct bactericidal activity, whereas MVs secreted from the *Staphylococcus hominis* strain S34-1 mediate potent antimicrobial competition via MP1 bacteriocin delivery [32].

Next, MRSA USA300 was treated with various concentrations of VAN@^Δagr^MVs (0, 6.25, 12.5, 25, and 50 μg/ml). After culturing at 37 °C for 12 or 24 h, the viable plate count revealed that VAN@^Δagr^MVs killed USA300 in a dose-dependent manner (Fig. 2C). Notably, treatment with 12.5 μg/ml VAN-loaded ^Δagr^MVs for 12 h substantially decreased the *S. aureus* cell load (Fig. 2C and D). Bacterial inactivation was further visualized by staining the treated *S. aureus* cells with a LIVE/DEAD backlight bacterial viability kit. Confocal laser scanning microscopy (CLSM) revealed that after 6 h of treatment, both 10 μg/ml of free VAN and 50 μg/ml of AN@^Δagr^MVs induced substantial cell death, whereas ^Δagr^MVs (50 μg/ml) had minimal effects comparable with those of the PBS control (Fig. 2E). Collectively, these results demonstrate that VAN encapsulated in ^Δagr^MVs preserves their intrinsic antibacterial activity and that VAN@^Δagr^MVs exhibit potent, dose-dependent efficacy against planktonic MRSA in vitro.

### ^Δagr^MVs-mediated penetration of VAN into MRSA biofilms

**Fig. 3.**
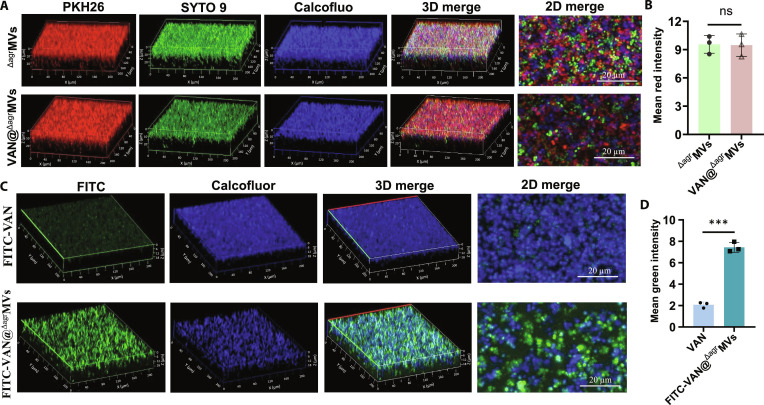
^Δagr^MVs-mediated penetration of VAN into MRSA biofilms. (A) CLSM revealed the penetration of ^Δagr^MVs and VAN@^Δagr^MVs into MRSA biofilms. Red fluorescence indicates PKH26-labeled ^Δagr^MVs, green fluorescence indicates SYTO 9-stained MRSA cells, and blue fluorescence represents the Calcofluor-stained biofilm matrix. (B) Mean red fluorescence intensity of ^Δagr^MVs and VAN@^Δagr^MVs in MRSA biofilms. (C) CLSM observation of FITC-VAN and FITC-VAN@^Δagr^MVs in MRSA biofilms. (D) Mean green fluorescence intensity of FITC-VAN and FITC-VAN@^Δagr^MVs penetrating biofilms. The data in (B) and (D) are shown as the mean ± SD. Statistical significance was measured by Student’s *t* test, ***P < 0.001, and ns represents no significance.

Bacterial biofilms represent complex communities of bacteria that are embedded in a self-produced ECM composed of polysaccharides, proteins, and eDNA [5]. This enriched matrix protects bacteria from environmental threats such as phage infection and antibiotic exposure [9]. Biofilm formation is a primary driver of drug resistance and recurrent infections [33]. Studies have shown that bacterial MVs play an important role in biofilm formation [34]; however, the ability of exogenous MVs to penetrate into mature biofilms remains poorly characterized. To achieve this, we first established *S. aureus* USA300 biofilms in confocal dishes (24 h culture) and then treated them with PKH26-labeled ^Δagr^MVs or VAN@^Δagr^MVs (red fluorescence) for 90 min at 37 °C. MRSA cells and the biofilm matrix were prestained with SYTO 9 (green) and Calcofluor White (blue), respectively [26]. CLSM revealed efficient penetration of both ^Δagr^MVs and VAN@^Δagr^MVs throughout the biofilm architecture, as demonstrated by 3-dimensional reconstruction and 2-dimensional cross-sectional analysis (Fig. 3A and Fig. S3). Fluorescence intensity analysis revealed comparable distributions of ^Δagr^MVs and VAN@^Δagr^MVs in MRSA biofilms (Fig. 3B). These findings demonstrate that VAN@^Δagr^MVs and ^Δagr^MVs exhibit homotypic targeting capacity for *S. aureus* biofilms and that the distributed VAN@^Δagr^MVs may inactivate bacteria via VAN release.

To specifically track antibiotic distribution, we labeled free VAN with FITC. The resulting FITC-VAN was encapsulated in ^Δagr^MVs to generate FITC-VAN@^Δagr^MVs. CLSM analysis revealed that free FITC-VAN failed to penetrate into the biofilm matrix (Fig. 3C). In contrast, FITC-VAN@^Δagr^MVs substantially accumulated within the biofilms (Fig. 3D and S4). These findings confirm that ^Δagr^MVs serve as effective delivery vehicles for enhanced antibiotic penetration into biofilms. Zavan et al. [35] reported that the autotransporter Ag43-containing MVs derived from *E. coli* markedly increase biofilm development, indicating the impact of specific MV compositions on bacterial aggregation and biofilm formation. However, the specific factors contributing to the ability of ^Δagr^MVs to mediate VAN penetration into biofilms are unclear. Identifying these factors will be crucial for developing optimized drug delivery systems against biofilm-associated infections.

### Destruction of biofilms by VAN@^Δagr^MVs in vitro

**Fig. 4.**
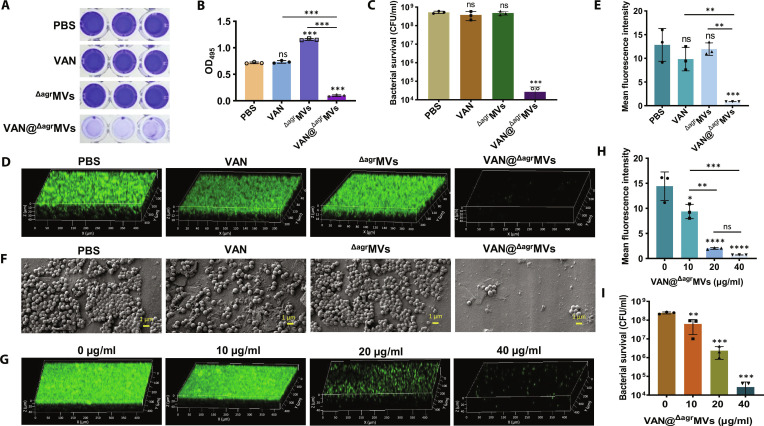
Antibiofilm activity of VAN@^Δagr^MVs. (A) Crystal violet staining of MRSA biofilms treated with VAN@^Δagr^MVs. PBS, free VAN, and empty ^Δagr^MVs served as controls. (B) OD_595_ values of crystal violet-stained biofilms after treatment with PBS, VAN, ^Δagr^MVs, and VAN@^Δagr^MVs. (C) Bacterial survival in biofilms treated with PBS, VAN, ^Δagr^MVs, or VAN@^Δagr^MVs. (D) CLSM observation of VAN@^Δagr^MVs-treated biofilms. PBS, VAN, and ^ΔagrA^MVs served as controls. Green fluorescence indicates SYTO 9-stained MRSA USA300 cells. (E) Mean fluorescence intensity of biofilms treated with various agents as indicated. (F) SEM image of VAN@^Δagr^MVs-treated biofilms. (G) CLSM observation of MRSA biofilms treated with various concentrations of VAN@^Δagr^MVs. (H) Mean fluorescence intensity of biofilms treated with various concentrations of VAN@^Δagr^MVs. (I) Bacterial survival in biofilms treated with various concentrations of VAN@^Δagr^MVs. The data are shown as the mean ± SD or standard error of the mean. Statistical significance was measured by 1-way ANOVA, **P < 0.01, ***P< 0.001, ****P< 0.0001, and ns represents no significance.

Bacterial biofilms, as surface-associated microbial communities, represent a major cause of recurrent infections in clinical settings [36]. While microbial growth inhibition is crucial for biofilm eradication [6], effective penetration of antimicrobial agents remains a key challenge. To investigate the biofilm eradication ability of VAN@^Δagr^MVs, we established *S. aureus* USA300 biofilms in 96-well plates or confocal dishes (24 h culture). After the free cells were washed with PBS, the mature biofilms were treated with PBS (control), free VAN (10 μg/ml), ^Δagr^MVs (50 μg/ml), or VAN@^Δagr^MVs (50 μg/ml) at 37 °C for 24 h. Crystal violet staining was performed as described previously [20], and the results revealed that VAN@^Δagr^MVs substantially eradicated biofilms compared with the controls (*P* < 0.001), whereas free VAN did not (Fig. 4A and B). Notably, treatment with ^Δagr^MVs alone markedly enhanced biofilm formation, although viable cell counts remained comparable to those of the PBS control (Fig. 4C and Fig. S5A). These results indicate that ^Δagr^MVs-mediated VAN delivery enables effective VAN penetration and biofilm removal, overcoming the limited accessibility of free VAN. Several studies have reported that the MVs of *Streptococcus mutans* carry diverse types of glucosyltransferases, therefore promoting biofilm formation [37]. With atmospheric SEM, Takahashi et al. [38] precisely showed that the released MVs are the first factor involved in stimulating biofilm establishment of *Staphylococcus epidermidis*. Fong et al. [39] demonstrated that key proteins such as RmbA and Bap1 loaded in *Vibrio cholerae* MVs play important roles in biofilm stability and formation. While our study demonstrated the critical role of ^Δagr^MVs in VAN delivery, the specific MV components responsible for biofilm modulation in *S. aureus* require further characterization. This knowledge will be essential for optimizing nanoparticle-based strategies against biofilm-associated infections.

Following established biofilm experimental guidelines [40], we assessed biofilm disruption by staining *S. aureus* with SYTO 9 and analyzing biofilm architecture via CLSM. Compared with those in the PBS, VAN@^Δagr^MVs resulted in substantially greater biofilm disruption (Fig. 4D and E). SEM further confirmed these findings, demonstrating nearly complete biofilm elimination following VAN@^Δagr^MVs treatment (Fig. 4F). However, VAN-treated samples presented some cellular disruption, and this effect was transient, likely due to limited antibiotic penetration during SEM sample preparation.

To evaluate the concentration-dependent effects, mature MRSA USA300 biofilms were treated with VAN@^Δagr^MVs (0 to 40 μg/ml) for 24 h. CLSM imaging coupled with SYTO 9 staining revealed a progressive, dose-dependent reduction in the number of viable bacteria (Fig. 4G and H). The corresponding plate counts demonstrated that 10 μg/ml of VAN@^Δagr^MVs significantly reduced bacterial load in the biofilms and that higher concentrations (20 to 40 μg/ml) increased bactericidal activity (Fig. 4I and Fig. S5B). Overall, these data demonstrate that, compared with conventional VAN treatment, ^Δagr^MVs-mediated VAN delivery overcomes penetration barriers in mature biofilms, exhibits dose-dependent antibacterial efficacy, and achieves superior biofilm eradication.

### Potential antibiofilm mechanisms of VAN@^Δagr^MVs

**Fig. 5.**
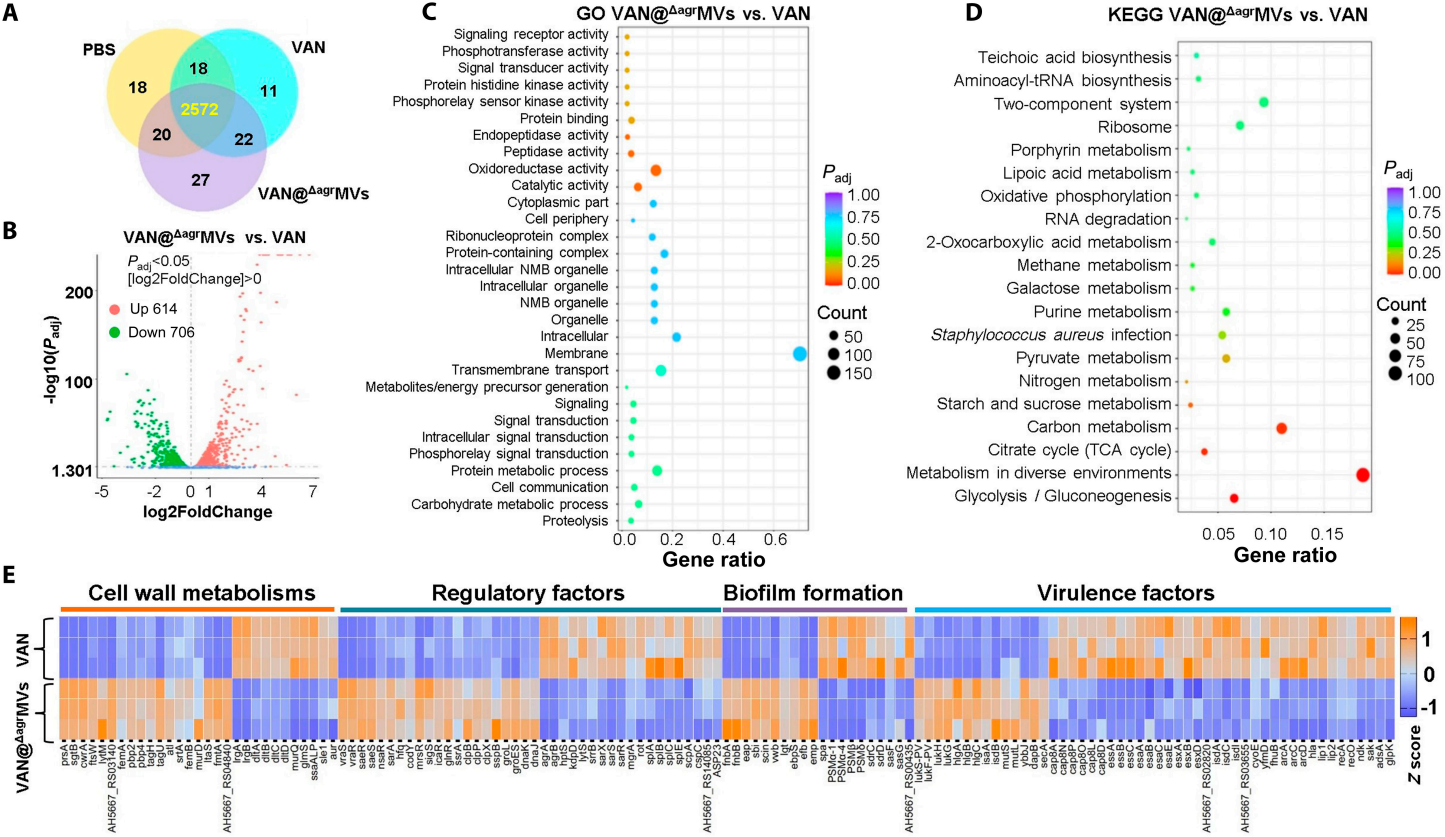
RNA-seq analysis of MRSA biofilms treated with VAN@^Δagr^MVs. (A) Schematic representation of differentially expressed genes (DEGs) in USA300 biofilms treated with PBS (yellow), free VAN (green), or VAN@^Δagr^MVs (purple). (B) DEGs in biofilms treated with VAN@^Δagr^MVs vs. VAN. (C) GO enrichment analysis of DEGs between VAN@^Δagr^MVs- and VAN-treated biofilms. (D) KEGG pathway analysis of DEGs between VAN@^Δagr^MVs- and VAN-treated biofilms. (E) Heatmap showing the transcriptional profiles of the indicated genes in each sample (*Z* score normalized).

VAN exerts its antimicrobial action by binding to the D-alanyl–D-alanine termini of lipid II, thus inhibiting peptidoglycan synthesis through disruption of transpeptidation and transglycosylation mediated by penicillin-binding proteins [41]. This interaction ultimately leads to cell wall degradation and bacterial lysis [42]. To explore the potential mechanisms underlying biofilm eradication by VAN@^Δagr^MVs, we performed transcriptomic analysis of MRSA USA300 biofilms following 3 h of treatment with PBS, free VAN, or VAN@^Δagr^MVs. RNA-seq revealed a total of 2,572 coexpressed genes across all groups (Fig. 5A). Notably, VAN@^Δagr^MVs treatment induced distinct expression profiles compared with those of the controls (Fig. S6A). Free VAN treatment up-regulated only *vraX* (Fig. S6B), a cell wall stress response gene associated with glycopeptide resistance [43]. These results are consistent with previous findings that VAN has a limited capacity to penetrate into bacterial biofilms [44].

RNA-seq analysis also showed that, compared with VAN alone, VAN@^Δagr^MVs treatment resulted in the expression of 1,320 DEGs (614 up-regulated, 706 down-regulated; *P* < 0.05 and |fold change| > 1.5) in MRSA USA300 (Fig. 5B). Gene Ontology (GO) enrichment analysis demonstrated substantial inhibition of genes involved in membrane and transmembrane transport functions, intracellular metabolic processes, and oxidoreductase activity (Fig. 5C). Similar gene patterns regarding membrane, cell, cell part, intracellular, oxidoreductase, and transporter activities were observed in bacteria treated with VAN@^Δagr^MVs compared with those in the PBS control (Figs. S6C and S7A). Moreover, Kyoto Encyclopedia of Genes and Genomes (KEGG) enrichment revealed that VAN@^Δagr^MVs primarily affected microbial metabolism pathways, carbon metabolism, 2-component systems, and ribosome and glycolytic pathways (Fig. 5D and Fig. S7B).

The stress-protecting cell wall of *S. aureus* is characterized by a thick peptidoglycan composed of repeating β-(1–4)-linked N-acetylglucosamine and N-acetylmuramic acid (MurNAc) disaccharide units. Each MurNAc residue is modified by a stem pentapeptide (L-Ala–D-Gln–L-Lys–D-Ala–D-Ala), with pentaglycine bridges connecting the ε-amino group of L-Lys to the D-Ala carboxyl group of adjacent peptides, creating an extensively cross-linked peptidoglycan network [41,42]. RNA-seq analysis revealed marked transcriptional changes in cell wall-related genes following VAN@^Δagr^MVs treatment, including up-regulation of peptidoglycan biosynthesis genes (*pbp2* and *pbp4*), increased expression of peptidoglycan metabolism regulators (*prsA*, *sgtB*, *cwrA*, and *atl*) (Fig. 5E), elevated *ltaS* expression (teichoic acid synthase), and down-regulation of the *dltABCD* operon, which mediates teichoic acid D-alanylation [45]. These findings demonstrate that ^Δagr^MVs-mediated VAN delivery induces profound cell wall stress or compensatory responses, disrupts teichoic acid modification pathways, and ultimately compromises bacterial structural integrity and virulence.

VAN is a cationic glycopeptide antibiotic originally isolated from *Amycolatopsis orientalis* (previously classified as *Streptomyces orientalis* or *Nocardia orientalis*) [42]. As a cell wall-targeting antibiotic, VAN-encapsulated ^Δagr^MVs treatment resulted in changes in a variety of regulatory factor genes, including the up-regulation of glycopeptide resistance-associated 2-component system *vraSR*, the virulence regulator *saeSR*, and the global regulators *sarA*, *hfq*, and *codY* (Fig. 5E and Fig. S8). These up-regulated genes might also be ascribed to the compensatory responses of MRSA under treatment with VAN@^Δagr^MVs. However, another important bacterial virulence regulator, the *agr* locus, was substantially reduced in *S. aureus* treated with VAN@^Δagr^MVs. Accordingly, an array of virulence factor genes, such as the α-hemolysin gene *hla*, capsule biosynthetic (*cap*) genes (*cap8A, cap8N, cap8P, cap8O, cap8L*, and *cap8D*), and type VII secretion system (T7SS) genes (*essA, essB, essC, esaA, esaB, esaC, esaE, esxA, esxB*, and *esxE*), were substantially down-regulated (Fig. 5E). α-Hemolysin (Hla) is a crucial virulence factor involved in *S. aureus* pathogenesis. Qian et al. [46] reported that artesunate inhibits staphylococcal biofilm formation by decreasing *hla* expression, suggesting a vital role of *hla* in *S. aureus* biofilms. A positive association between capsular polysaccharide and biofilm formation in *S. aureus* has been verified [47]. However, the functions of T7SS factors in the biofilm formation of *S. aureus* remain unclear. Our findings open a future direction for elucidating the role of T7SS apparatus components or their effectors in promoting *S. aureus* biofilm formation.

Additionally, several biofilm-related genes were up-regulated in *S. aureus* biofilms treated with VAN@^Δagr^MVs (Fig. 5E and Fig. S8C). The components of the biofilm ECM are complex. Studies have shown that the fibronectin-binding proteins FnBA and FnBB, extracellular adherence protein (Eap), immunoglobulin G-binding protein Sbi, staphylococcal complement inhibitor (SCIN), and clotting factor Vwb are the predominant ECM constituents [8]. These results showed that the antibiofilm effect of VAN@^Δagr^MVs could induce the compensatory responses of MRSA by increasing the expression of ECM molecules. Phenol-soluble modulins (PSMs) are vital toxins involved in bacterial biofilm dispersal [48]. Our results revealed that PSM toxins, including PSMα-1, PSMα-4, PSMβ, and PSMδ, were substantially inhibited in *S. aureus* USA300 after treatment with VAN@^Δagr^MVs (Fig. 5E). Moreover, the expression of SpA and the cell wall-anchored proteins SdrC and SdrD was also down-regulated after treatment with VAN@^Δagr^MVs. Graf et al. [49] cultivated *S. aureus* biofilms via a flow system and profiled their intracellular and extracellular biofilm proteomes. They found that many virulence factors in *S. aureus* biofilms are highly expressed, as indicated by the abundance of capsule biosynthesis proteins along with various secreted virulence factors, including hemolysins, leukotoxins, and lipases, as part of the ECM. Our data further support their conclusion. Overall, these results indicate that VAN@^Δagr^MVs can specifically reduce capsule biosynthesis and T7SS function by affecting their regulators.

### Antibiofilm activity of VAN@^Δagr^MVs in vivo

**Fig. 6.**
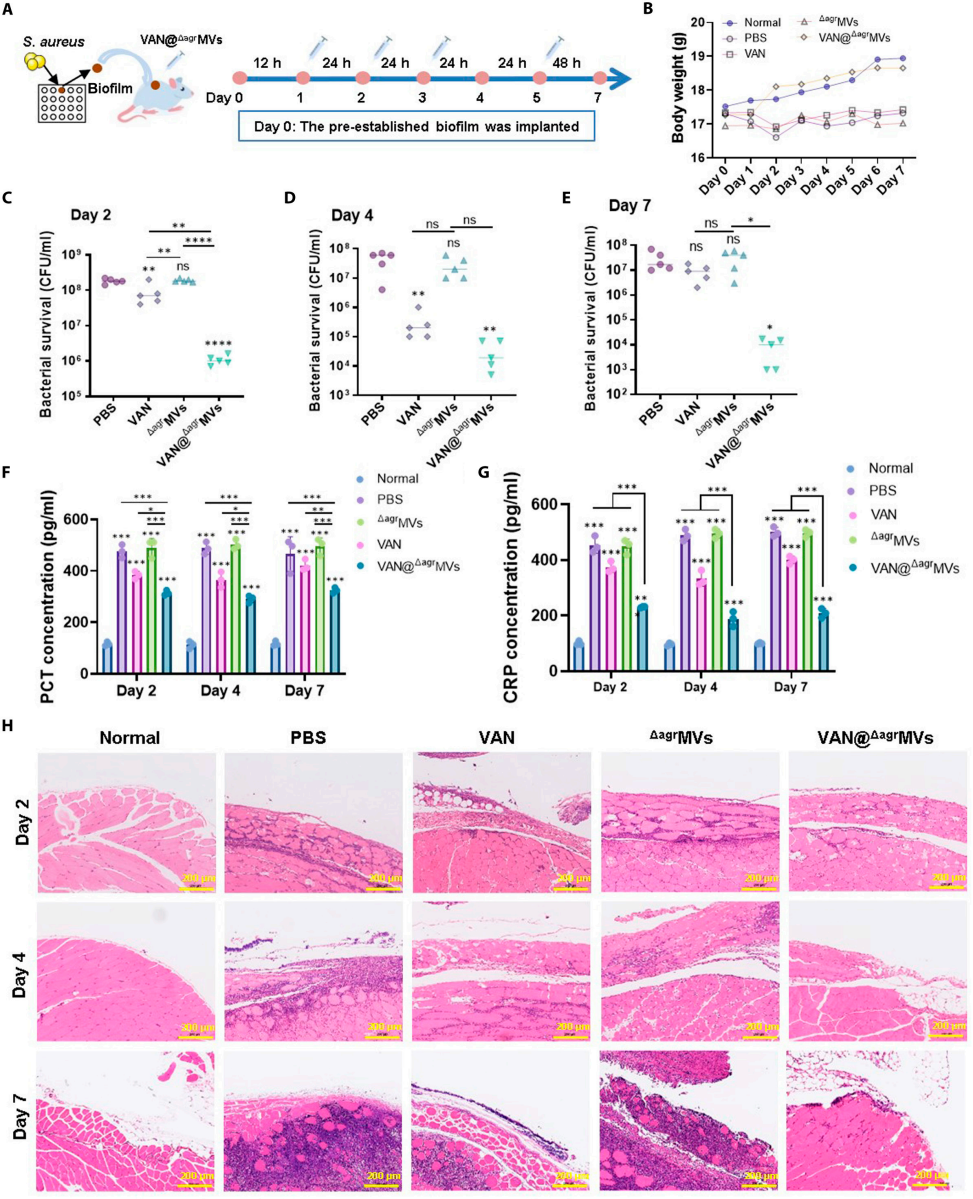
Antibiofilm efficacy of VAN@^Δagr^MVs in vivo. (A) Schematic showing the construction and treatment of a biofilm-infected murine model. MRSA USA300 biofilms grown on silicone sheets were subcutaneously implanted into BALB/c mice (left). The murine model received 4 doses of treatment within 7 days (right). (B) The weight changes of mice within 7 days. (C to E) Bacterial counts in biofilms after treatment with PBS, VAN, ^Δagr^MVs, or VAN@^Δagr^MVs on days 2 (C), 4 (D), and 7 (E) (*n* = 5 for each time point). (F) PCT and (G) CRP levels in biofilm-infected mice on days 2, 4, and 7 (*n* = 3). (H) Histopathological examination of murine skin tissues around the biofilm implantation site after H&E staining. The data are shown as the mean ± SD. Statistical significance was measured by 1-way or 2-way ANOVA, *P < 0.05, **P < 0.01, ***P < 0.001, ****P < 0.0001, and ns represents no significance.

To confirm the biofilm elimination capacity of VAN@^Δagr^MVs, we generated a subcutaneous biofilm-infected murine model by implanting a preestablished biofilm on a silicone sheet into the dorsal subcutis (Fig. 6A and Fig. S9). The mice were treated with PBS, VAN (2 μg), ^Δagr^MVs (10 μg), or VAN@^Δagr^MVs (10 μg, containing 2 μg VAN) after 12 h of biofilm implantation, followed by treatment on days 2, 3, and 5 to assess sustained biofilm clearance and recurrence prevention (Fig. 6A). Body weighting revealed decrease of mouse weights in PBS-, VAN-, and ^Δagr^MVs-treated groups, while VAN@^Δagr^MVs treatment restored mouse growth comparable to the normal control (Fig. 6B). Moreover, bacterial counts from colonized silicone implants were performed on days 2, 4, and 7 during treatment. The results showed that VAN treatment substantially reduced the *S. aureus* burden on days 2 and 4 compared with those in the PBS and ^Δagr^MVs groups (Fig. 6C and D and Fig. S10), while exhibiting comparable bacterial load on day 7 (Fig. 6E). The treatment with ^Δagr^MVs only did not show therapeutic effect on MRSA biofilms; however, VAN@^Δagr^MVs administration achieved the lowest bacterial load among all treatment groups on days 2, 4, and 7 (*P* < 0.05; Fig. 6C to E), demonstrating the effective biofilm elimination capacity of VAN@^Δagr^MVs in vivo. Although gradually reduced bacterial burden was presented with the duration of VAN@^Δagr^MVs treatment, low quantity of bacteria survived at the end of experiment (day 7; Fig. 6E), suggesting a longer-term treatment needed for complete eradication of MRSA biofilms in vivo. As for this phenomenon, the potential for *S. aureus* cells to develop resistance against VAN@^Δagr^MVs could happen within biofilms. In addition, the specific mechanisms underlying preferential uptake of VAN@^Δagr^MVs by isogenic bacteria need further investigation.

Recent studies have indicated that inflammatory markers such as C-reactive protein (CRP) and calcitoninogen (PCT) correlate with bacterial infections and serve as diagnostic indicators [50]. ELISA analysis of murine sera revealed that, compared with the uninfected control (normal group), *S. aureus* biofilm infection substantially increased PCT and CRP levels (PBS treatment vs. normal group). While ^Δagr^MVs alone did not reduce the levels of these elevated markers, both VAN and VAN@^Δagr^MVs decreased PCT and CRP levels compared with those in the PBS control on days 2, 4, and 7 (Fig. 6F and G). VAN@^Δagr^MVs-treated mice presented the lowest marker levels among all treatment groups, although these levels remained higher than those of the normal control, potentially because of residual bacteria. Moreover, histopathological examination of biofilm-infected skin tissues revealed increased inflammatory cell infiltration in the groups of PBS, VAN, and ^Δagr^MVs from day 2 to day 7; however, the treatment with VAN@^Δagr^MVs resulted in the continuous decline of skin inflammation (Fig. 6H). Overall, these results demonstrate that VAN@^Δagr^MVs effectively reduce the bacterial load in established biofilms and attenuate infection-associated inflammatory responses.

### Safety of VAN@^Δagr^MVs

**Fig. 7.**
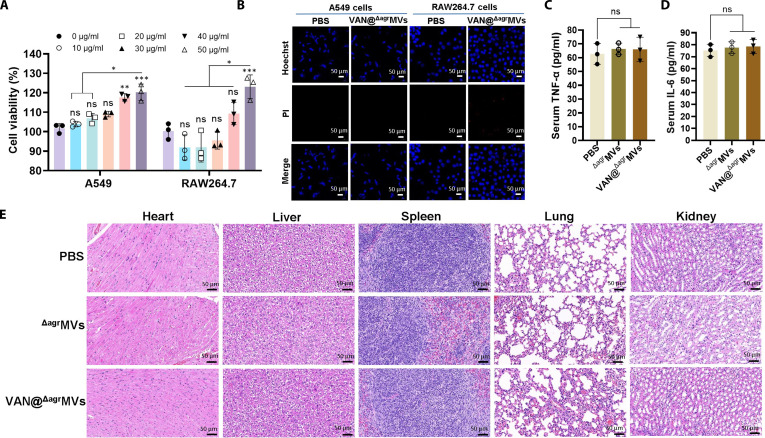
Safety assessment of VAN@^Δagr^MVs in vitro and in vivo. (A) CCK-8 assay for determining the viability of A549 and RAW264.7 cells treated with various concentrations of VAN@^Δagr^MVs. (B) LIVE/DEAD cell staining of A549 and RAW264.7 cells treated with 50 μg/ml of VAN@^Δagr^MVs. (C) TNF-α and (D) IL-6 levels in BALB/c mice injected with PBS, ^Δagr^MVs, or VAN@^Δagr^MVs for 24 h. (E) Histopathological examination of murine organ tissues via H&E staining. The data are shown as the mean ± SD. Statistical significance was measured by 1-way or 2-way ANOVA, *P < 0.05, **P < 0.01, ***P < 0.001, and ns represents no significance.

As a promising antibiofilm candidate, the potential toxicity of VAN@^Δagr^MVs is a primary concern for future applications [9]. To evaluate cytotoxicity in vitro, we used alveolar epithelial type II cells (A549) and mouse RAW264.7 macrophages. Cell Counting Kit (CCK)-8 assays revealed that treatment with 50 μg/ml VAN@^Δagr^MVs had no effect on either cell type, with substantially increased viability observed after 24 h of coincubation (Fig. 7A). LIVE/DEAD cell staining further confirmed these results, revealing comparable numbers of viable A549 and RAW264.7 cells between the VAN@^Δagr^MVs-treated group and the PBS control after 24 h (Fig. 7B). These findings align with our previous report that deletion of the *agr* locus substantially reduces the potential toxicity of *S. aureus* MVs [16].

We next evaluated the toxicity of VAN@^Δagr^MVs in vivo. BALB/c mice received intraperitoneal injections of PBS, ^Δagr^MVs, or VAN@^Δagr^MVs. After 24 h, blood samples were collected for inflammatory factor detection. The results revealed comparable serum IL-6 and TNF-α levels among the VAN@^Δagr^MVs-treated, ^Δagr^MV-administered, and PBS control groups (Fig. 7C and D). Histopathological examination of heart, liver, spleen, lung, and kidney tissues revealed normal morphology in all groups, as demonstrated by H&E staining (Fig. 7E). Taken together, these findings indicate that VAN@^Δagr^MVs represent a safe formulation for treating biofilm-associated infections.

## Conclusion

**Fig. 8.**
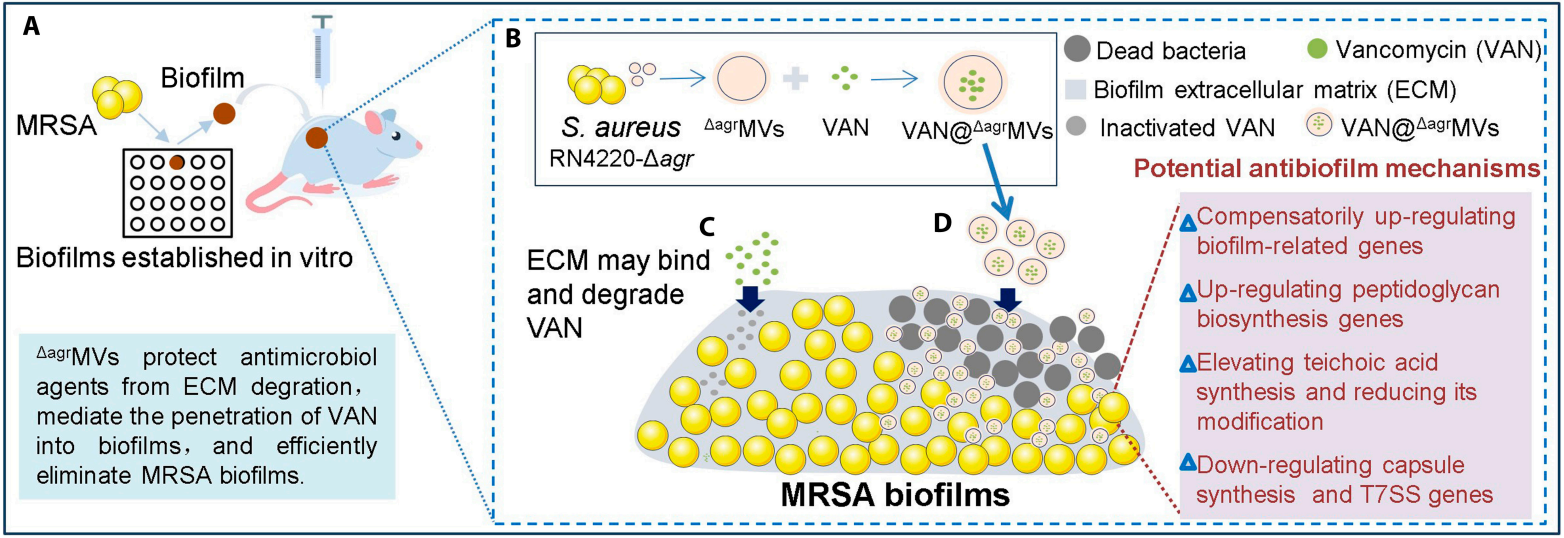
Schematic summary of the antibiofilm activity of VAN@^Δagr^MVs. (A) Construction and treatment of MRSA biofilm-infected murine model. (B) Preparation of VAN@^Δagr^MVs. (C) Free VAN can be degraded by biofilm ECM. (D) ^Δagr^MVs-mediated penetration of VAN to eliminate MRSA biofilms via diverse potential mechanisms.

In summary, we utilized attenuated *S. aureus* ^Δagr^MVs to encapsulate VAN for biofilm elimination (Fig. 8). ^Δagr^MVs effectively delivered VAN, and VAN@^Δagr^MVs were easily prepared. The VAN@^Δagr^MVs improved VAN bioavailability and exhibited sustained release. Unlike free VAN treatment alone, the VAN@^Δagr^MVs treatment considerably eradicated MRSA USA300 biofilms, as evidenced by the superior penetration of the nanoparticles into the biofilms and substantial reduction of the MRSA burden in biofilms. The antibiofilm mechanism likely involves combined cell wall inhibition and biofilm matrix disruption. In a biofilm implantation model, VAN@^Δagr^MVs treatment resulted in the lowest bacterial load among all the experimental groups. Both in vitro and in vivo safety assessments confirmed the biocompatibility of VAN@^Δagr^MVs, supporting their potential clinical application. Overall, this study presents a novel strategy against MRSA biofilm infections and offers a rational framework design for isogenous antibiofilm nanomaterials.

## Ethical Approval

All procedures involving animals were conducted in compliance with the Guidelines for the Care and Use of Laboratory Animals at Army Medical University and were approved by the Animal Ethics Committee of Army Medical University (Protocol no. AMUWEC2020735).

## Data Availability

All data that support the findings of this study are available upon request from the corresponding authors. The RNA-seq data have been deposited in the SRA datasets under the ID code PRJNA1279791.
