# Supplementary material for: Homotypic Membrane Vesicle-Formulated VAN@ΔagrMVs for Methicillin-Resistant Staphylococcus aureus Biofilm Clearance
Source: Biomater Res. 2025 Dec 9;29:0288. doi: 10.34133/bmr.0288 (PMC12686342; doi:10.34133/bmr.0288)
Supplement: Supplementary 1 — Figs. S1 to S10 [file bmr.0288.f1.docx]

**Homotypic membrane vesicle-****formulated VAN@^Δagr^MVs for methicillin- resistant *Staphylococcus aureus* biofilm clearance**

Jianxiong Dou ^#^, Huagang Peng ^#^, Shu Li ^#^, Weilong Shang, Yi Yang, Xiaomei Hu, Li Tan, Zhen Hu, Yuting Wang, Feng Lin, Qiwen Hu, Chuan Xiao, Xiaoran Jiang, Ming Li*, and Xiancai Rao*

Department of Microbiology, College of Basic Medical Sciences, Army Medical University, Key Laboratory of Microbial Engineering under the Educational Committee in Chongqing, Chongqing, 400038, China.

^#^ These authors contributed equally to this work.

E-mail:

* Correspondence:

Xiancai Rao, raoxiancai@126.com, or xcrao@tmmu.edu.cn;

Ming Li, liming@tmmu.edu.cn


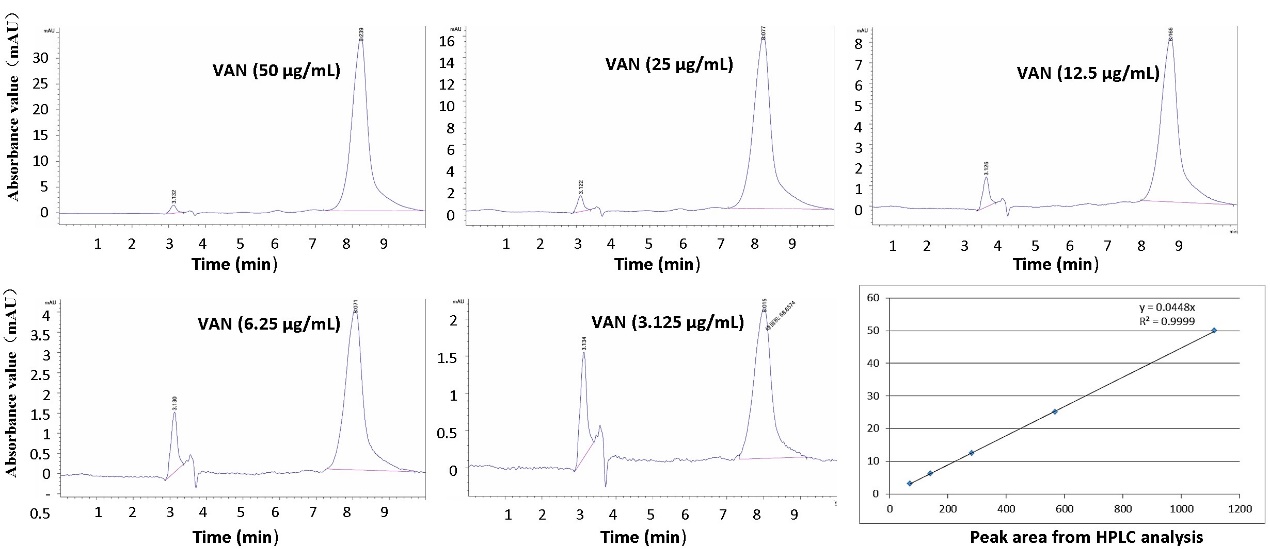


**Fig. S1.** The standard curve of VAN in HPLC analysis.

**
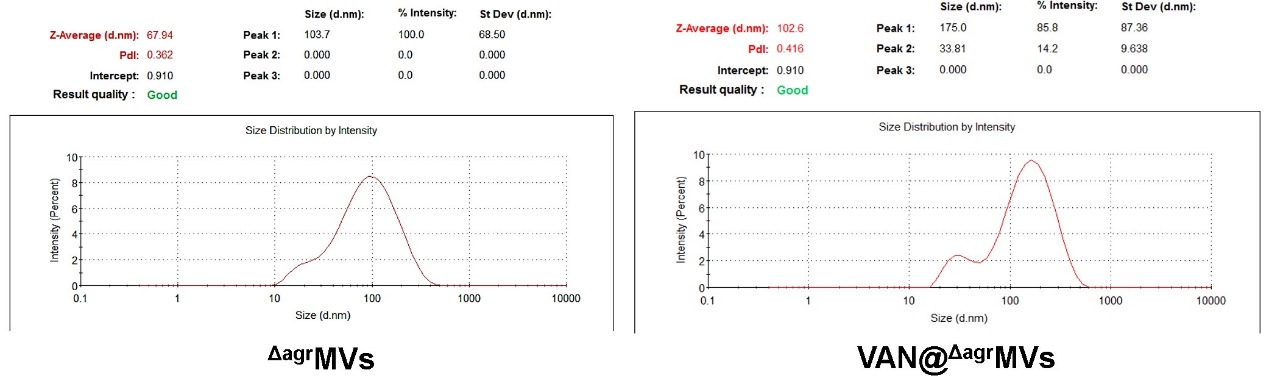
**

**Fig. S2.** Particle size distribution intensities of ^Δagr^MVs and VAN@^Δagr^MVs tested via DLS.


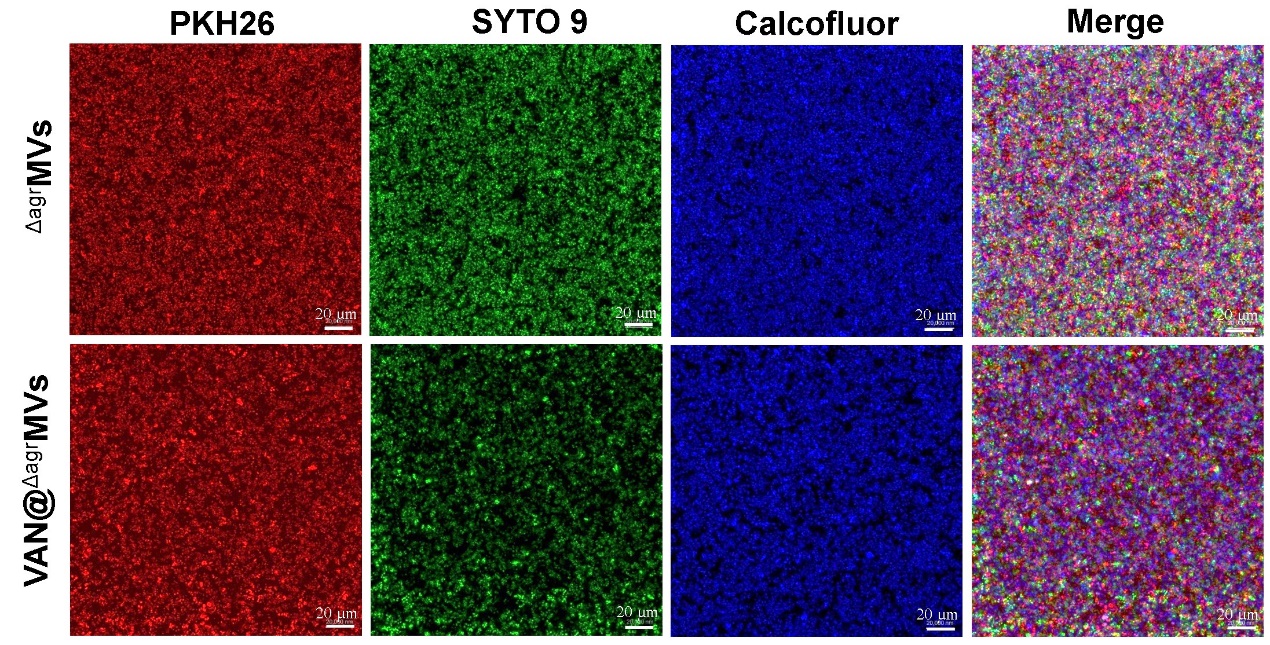


**Fig. S3.** 2D cross-sectional CLSM analysis of ^Δagr^MVs-mediated penetration of VAN into MRSA biofilms. Red fluorescence indicates PKH26-labeled ^Δagr^MVs, green fluorescence shows SYTO 9-stained MRSA cells, and blue fluorescence represents Calcofluor-stained biofilm matrix.


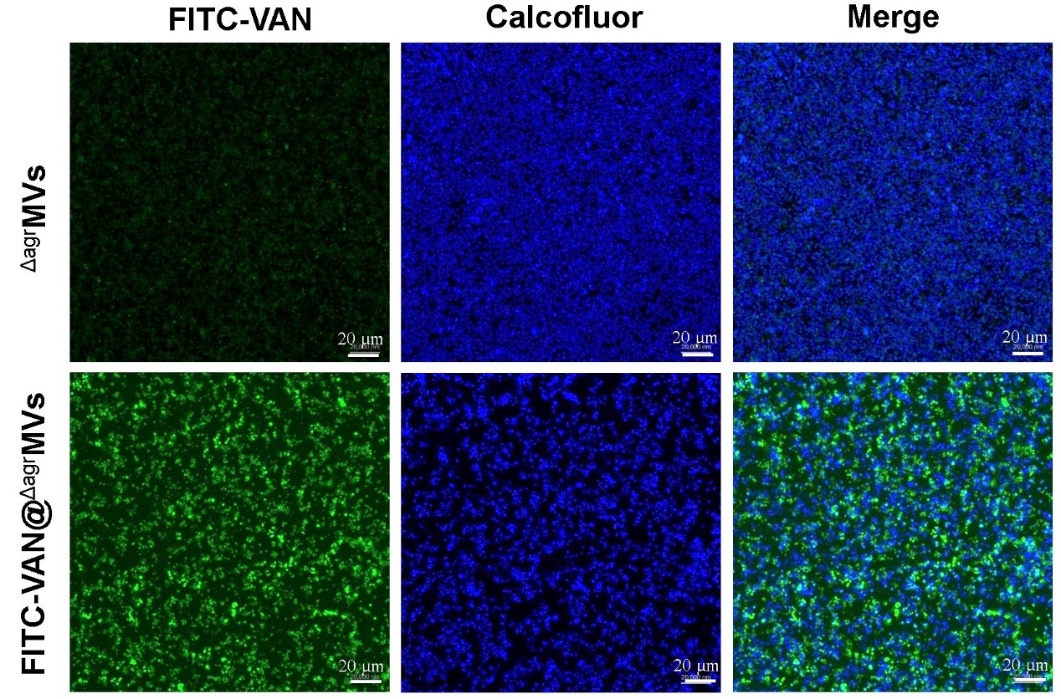


**Fig. S4.** CLSM observation of ^Δagr^MVs and FITC-VAN@^Δagr^MVs in MRSA biofilms. Green fluorescence shows FITC-labeled VAN and blue fluorescence represents Calcofluor-stained biofilm matrix.


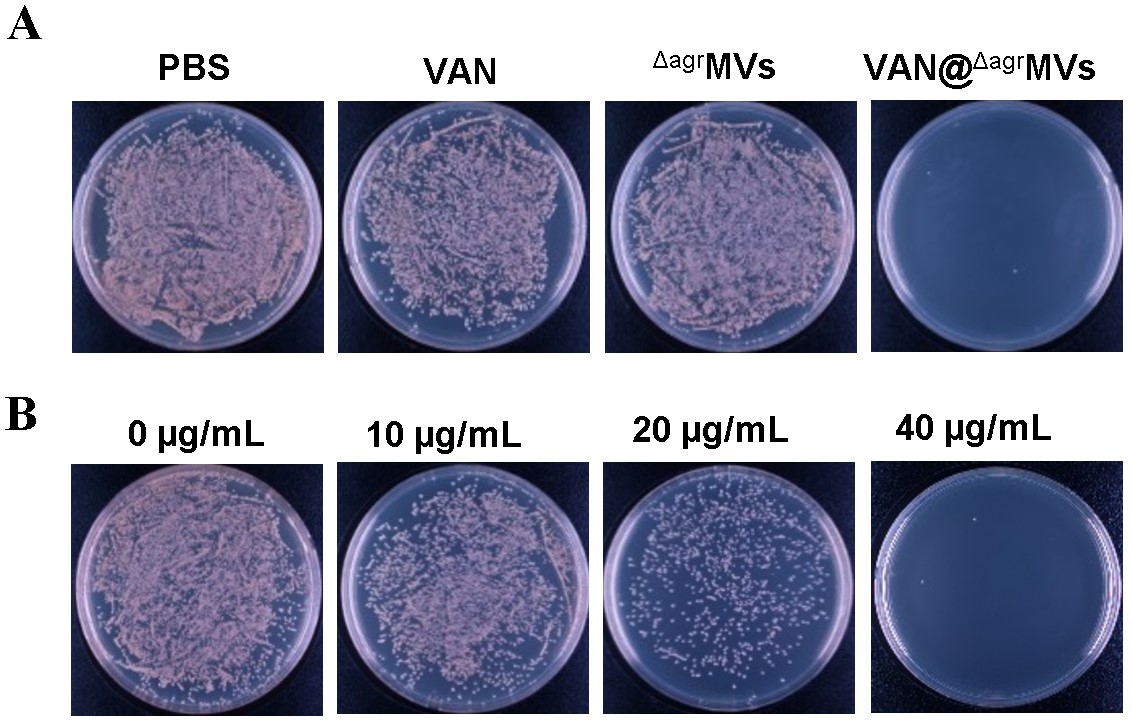


**Fig. S5.** Antibiofilm activity of VAN@^Δagr^MVs in vitro. (**A**) Growth of bacterial survivals derived from biofilms after treatment with PBS, VAN, ^Δagr^MVs, and VAN@^Δagr^MVs for 24 h. (**B**) Growth of MRSA USA300 survivals in biofilms treated with diverse concentrations of VAN@^Δagr^MVs for 24 h.


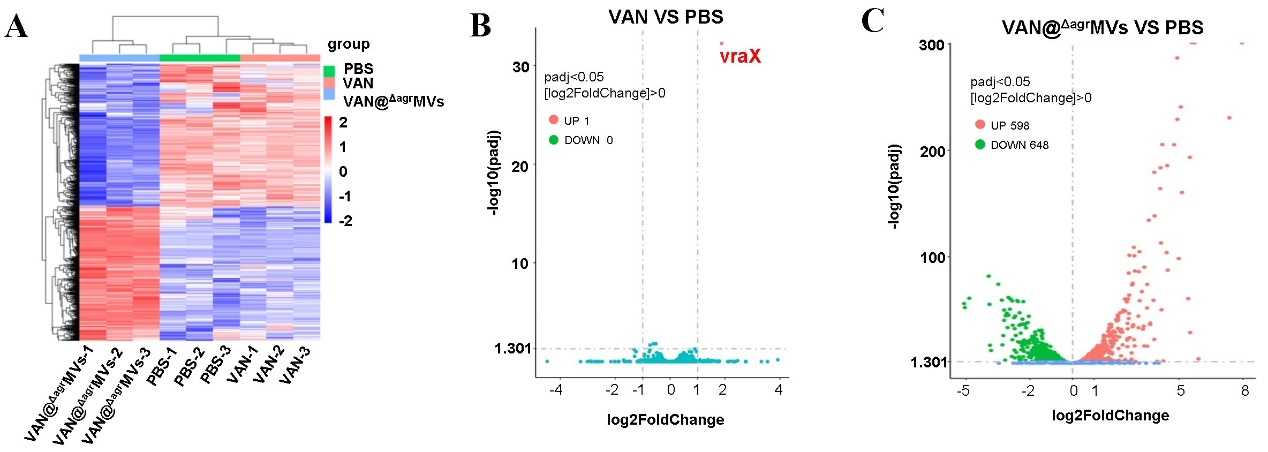


**Fig. S6.** RNA-seq analysis of MRSA biofilms treated with VAN@^Δagr^MVs for 3 h. (**A**) Heat map showing transcriptional profiles in each indicated sample analyzed with Pairwise Euclidean distance. (**B**) RNA-seq analysis showing DEGs in MRSA USA300 biofilms treated with VAN vs PBS. (**C**) RNA-seq analysis showing DEGs in MRSA USA300 biofilms treated with VAN@^Δagr^MVs vs PBS.


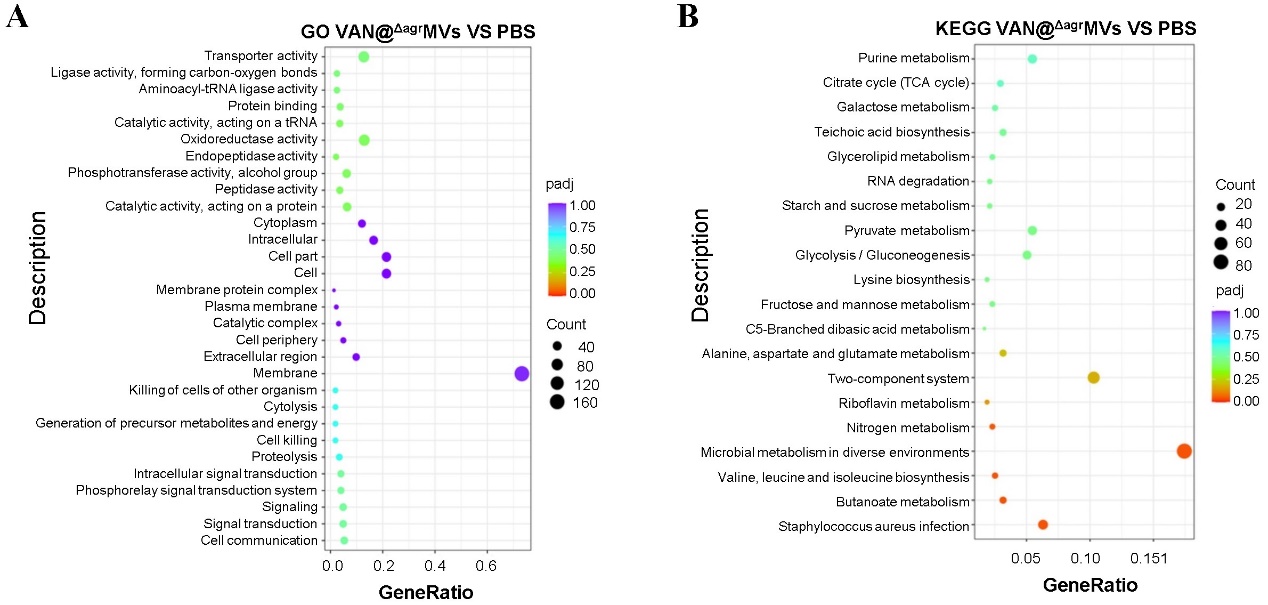


**Fig. S7.** Analysis of RNA-seq data. (**A**) GO analysis of DEGs between VAN@^Δagr^MVs- and PBS-treated MRSA biofilms. (**B**) KEGG analysis of DEGs between VAN@^Δagr^MVs- and PBS-treated MRSA biofilms.


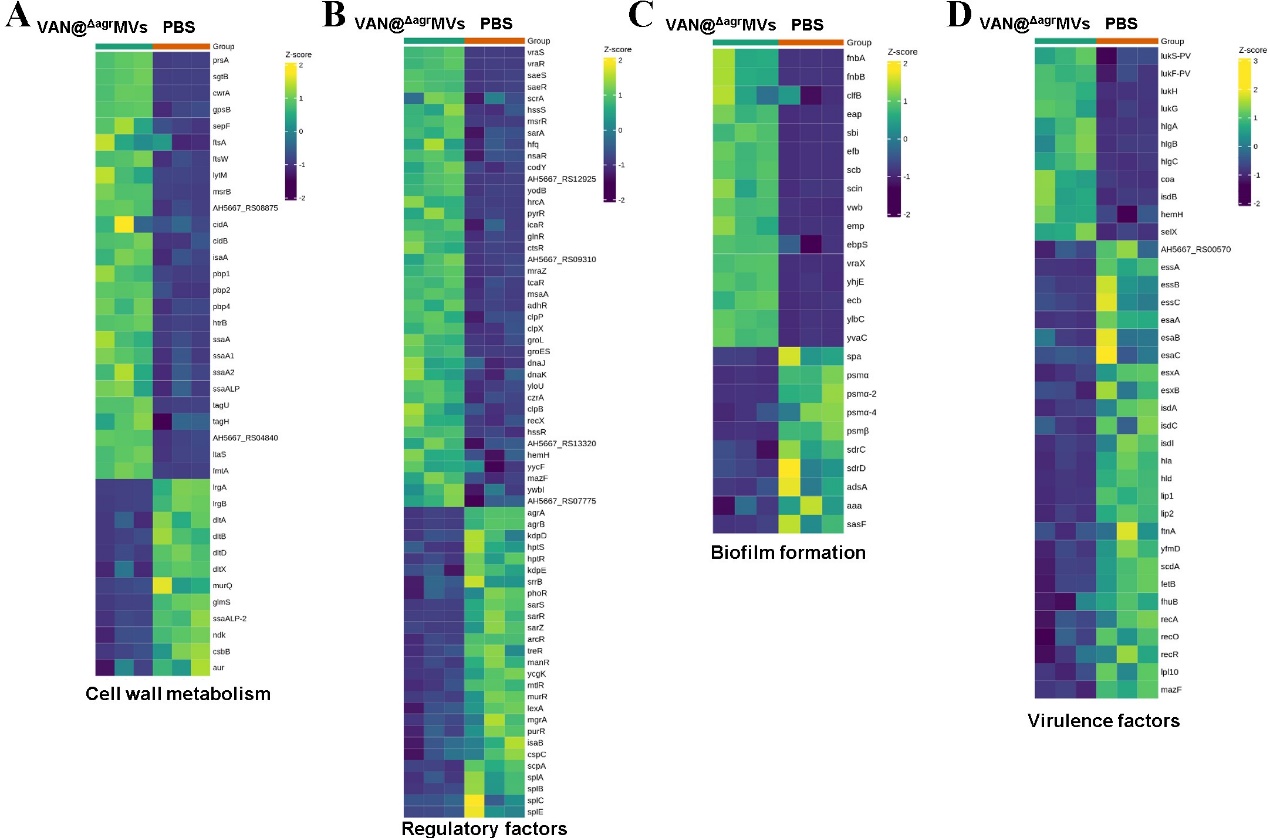


**Fig. S8.** Heat map showing transcriptional profiles of the indicated genes in each sample analyzed with normalized Z-score. (**A**) Cell wall metabolism associated genes. (**B**) Regulatory genes. (**C**) Biofilm formation associated genes. (**D**) Virulence factor genes.


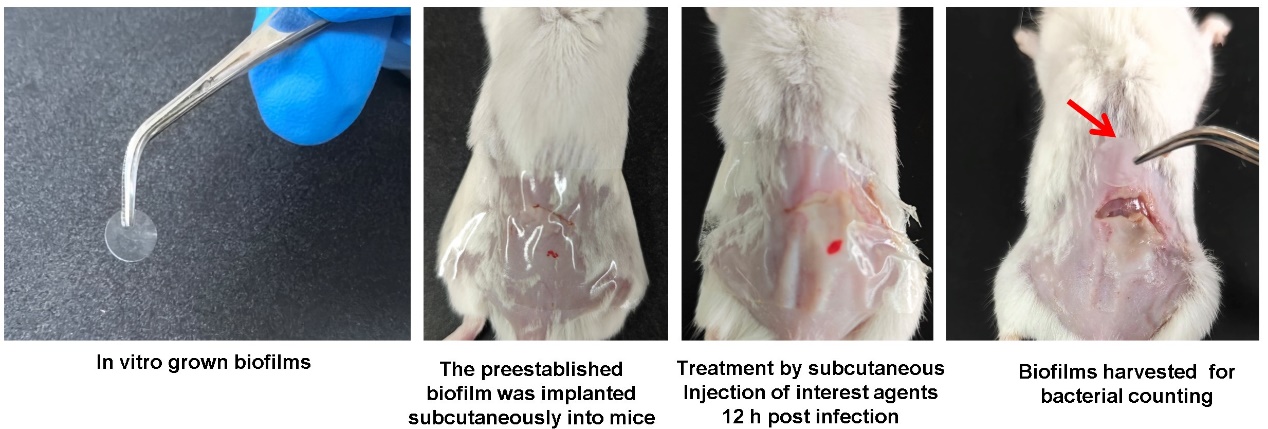


**Fig. S9.** Established a subcutaneous biofilm-infected murine model by implanting a silicone sheet with pre-established biofilms into the dorsal subcutis. The model was subjected to treatment (four times) with PBS, free VAN, ^Δagr^MVs, or VAN@^Δagr^MVs. Finaly, the biofilm was harvested for bacterial counting.


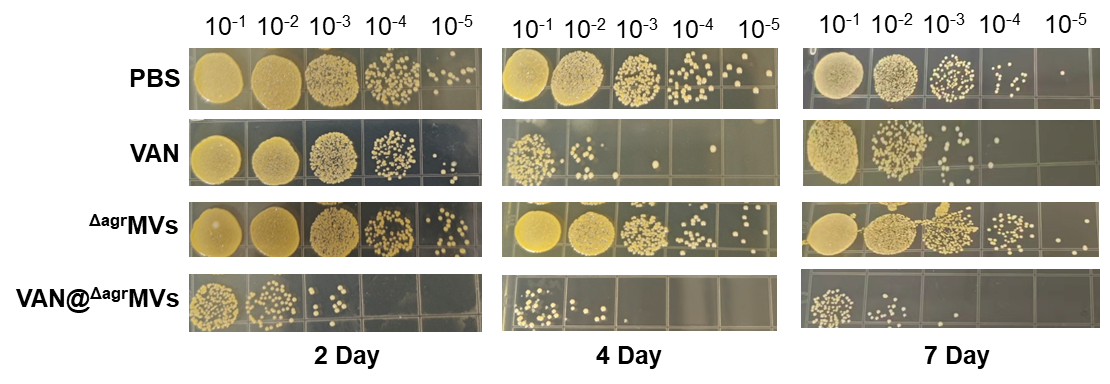


**Fig. S10.** Colony counts were performed on Days 2, 4, and 7 post-biofilm infection.
